# Association between trust in the public healthcare system and selecting a surgeon in public hospitals in Israel: a cross-sectional population study

**DOI:** 10.1186/s13584-020-00396-z

**Published:** 2020-07-27

**Authors:** Adi Niv-Yagoda

**Affiliations:** 1 Sackler Faculty of Medicine at Tel Aviv University, Tel Aviv, Israel; 2 School of Health Systems Management at Netanya Academic College, Netanya, Israel

**Keywords:** Public health system, Trust, Confidence, Surgeon selection, National Health insurance law, Regulation in health, Public hospital, Equality in health, Policy makers

## Abstract

**Background:**

The Israeli public health system has seen a steady decline in public trust and confidence, which has resulted in an increased rate of individuals holding private and commercial health insurance policies that allow more choice of various services (especially choose the surgeon’s). This study evaluated the attitudes and beliefs of Israeli adults regarding public trust, equitability and choice within the public health system.

**Methods:**

A cross-sectional telephone survey conducted among a representative random sample of Israeli adults (> 25 years). Participants responded to a 27-item questionnaire. Multivariate regression analyses were performed to determine the contribution of various socio-demographic variables to the perceptions of trust and equitability in the health system and the ability to choose a surgeon, As well as a possible links among these parameters.

**Results:**

Of 865 adults that responded to the survey, most were women (51.8%), Jewish (68.6%), and married (73.0%). Trust in the public health system, the perception of the system’s equitability and the public’s perception of the importance of selecting a surgeon were inter-related. The results emphasize a possible association between three meaningful factors: the trust in the public health system, the perception of the system’s equitability and the public’s perception regarding the importance of selecting a surgeon.

**Conclusions:**

Public trust in the public health system is a fundamental condition for maintaining an efficient and equitable health system in Israel. The survey suggests that uncertainty regarding the identity of the surgeon who will perform a procedure in a public hospital may be linked to a sense of insecurity and distrust of the public in the public health system. This study did not examine the causal relationship between the various factors, but the study data suggests a possible link between lower trust in the system and a lower perception of its equitability, and a subsequent associated increase in the public’s desire to select a surgeon. This study suggests to recognize public trust as a central and significant tool to strengthen public health system. One of the ways to strengthen the public’s confidence in the public health system could be to provide the patient with reliable information regarding parameters such as the identity of the senior surgeon in the operating room or the surgeon’s suitability for the patient’s medical condition.

## Introduction

In January 1995, the National Health Insurance Law came into effect in Israel [1]. This law redefined the healthcare services that permanent residents are entitled to receive from the public health system. Within this law, the state undertook to provide permanent residents with a basket of health services through health maintenance organizations (HMOs), independently of the individual’s ability to pay (In some situations there is a payment of deductible from the insured). The HMOs provide health services to the insured within their respective communities or within the HMOs’ network of affiliated hospitals. These services are provided in HMO clinics or through the purchase of services from external suppliers (for example: independent doctors, laboratories, medical Center’s and more), primarily hospitals, which account for the main HMO expenditures.

The law sought to break the link between the individual’s economic ability and the receipt of needed (i.e., medically justified) treatments, and all in accordance with the basket of medical services and the conditions defined by law. Services are provided by four competing HMOs (Clalit Health Services, Maccabi Healthcare Services, Leumit Health Care Services and Kupat Holim Meuhedet) to which an individual registers. Individuals can also switch from one HMO to another, under certain limits defined by the law. Members of each HMO have the freedom to choose between service providers, including physicians; however, since the resources of the public health system are limited, the law allows for some limits on this freedom of choice in order to enable HMOs and hospitals to plan and manage resources efficiently. Hence, according to the National Health Insurance Law, an HMO member is entitled to choose a service only from among a list of national or regional providers approved by the HMO or by the national health system. The law further stipulates that each HMO can determine the criteria for selecting between service providers and their scope in accordance with guiding criteria set out in the regulations [2]. These criteria must be transparent and available to the public. In recent years, and following policy changes, regulatory rules have been created that have also influenced the issue of choice (e.g. new rule requiring waiting period between seeing the same doctor in a public clinic and after in private).

The issue of the “scope of choice” within the public health system preoccupies all elements in the healthcare system, hinging on the axis between increased recognition of the individual’s autonomy and the desire to allow more patient choice within the system, and between recognizing the limitations of resources and the influence of choice on the realization of the principle of equitability and on the makeup of the public health system. This professional and public discussion has accompanied the health system since the beginning, intensifying over the years. Especially because studies indicate that the public in Israel believe it is important to choose hospitals and surgeon’s [3].

One of the main problems of the Israeli public health system is the steady decline in public trust and confidence in the public health system that may be attributed to various incentives, the lengths of public service queues, a sense of uncertainty and bureaucracy [4, 5].

One of the expressions of this trust decline was a sharp increase in the rate of individuals holding private and commercial health insurance policies, so that many Israeli residents have both the public health insurance they are entitled to by law and a commercial/private insurance [5, 6].

It was hypothesized that the public’s perception of the public health system as unequitable and untrustworthy nourishes the public’s sense of need to select their service provider, e.g., a surgeon, and therefore to hold private insurance, which allows greater choice of service providers. As a result, the public’s perception translates into large private health expenditure [6–11].

As a preliminary stage for this study, qualitative research was conducted among senior policy makers in the health care system. Semi-structured interviews were conducted with 27 senior managers and policy makers from the Ministry of Health; Ministry of Finance; health clinics; public hospitals; private hospitals; academia; insurance companies; organizations and associations. In-depth, semi-structured interviews, were used as a window for understanding and structuring processes from the point of view of this unique and essential group. The qualitative analysis was performed using the ‘Narralizer’ software that helps the researcher to encode the interviewed meaning units, and to identify and map patterns and categories. As part of integrative methodology, the qualitative research findings served as the basis for the quantitative survey which is the focus of this article.

The qualitative preliminary study found that, in the opinion of the policymakers interviewed, the public’s trust in the public system constitutes an essential factor for its functioning, and for the efficiency of the health system in Israel. Public trust was identified by the policymakers as having a major and significant impact on public behavior (purchasing insurance and turning to private medicine). They also indicated that the patients’ sense of uncertainty in the public health system as a factor that undermines trust. In the opinion of policymakers, it is the public’s confidence in the public health system that affects the feeling of having to choose a surgeon. The lower the confidence in the health system, the greater the patient’s desire to choose the surgeon’s identity [12].

In this quantitative study presented in this article, we evaluated the attitudes and beliefs of a representative sample of Israeli adults regarding trust in public health system, equitability and choice a surgeon’s within the public health system.

## Methods

### Study design and participants

A survey was conducted by telephone February 2018 among a random sample of adult Israelis (25 years of age and older), representing all segments of society. The participants’ contact details were was obtained from a national database by Geocartography Knowledge Group. Interviews were conducted, as needed, in Hebrew, Arabic or Russian by surveyors from Geocartography (Tel Aviv, Israel).

### Data collection

For this study we constructed a 27-item questionnaire to measure the attitudes and beliefs of the Israeli public regarding equatability and choice within the public health system. An initial pool of items was compiled based on consultation with psychometricians and experts in health policy and statistical research methods. The questionnaire included questions on participant demographics as well as statements to which the respondents had to indicate their agreement on a 4-point Likert scale (1, Agree to a very great extent, 2, Agree to a great extent, 3, Agree slightly, 4, Do not agree).

The questionnaire was chosen in order to provide an accurate comparison to the National questionnaire (Survey of Public Opinion on the Level of Service and Performance of the Healthcare System in Israel). To ensure the clarity of the statements and frequency of responses to each statement, a pilot study was initially conducted with 50 interviewees.

### Statistical analysis

The data collected in the questionnaires were typed and reviewed (checking the validity of “closed” variables). Then, a factor analysis was performed, combining the variables and creating a temporary summary score (index) that expressed the average obtained from all of the answers given on the same scale. Mean and standard deviation (SD) were calculated for each of the statements, as well as for groups of statements from the same area. In addition, alpha Cronbach reliability tests were performed followed by principle component analysis with varimax rotation. This rotation allows differentiating among the indices of the various factors.

Univariate and multivariate regression analyses were conducted to determine the socio-demographic variables (age, income, health status, education, gender, sector, marital status, HMO membership) contributing to “trust in the public health system”, “the perception of the health system as inequitable” and “the importance of selecting a surgeon”. Regression analysis was also performed to determine the contribution of “the perception of the health system as inequitable” and “trust in the public health system” to “the importance of selecting a surgeon”.

## Results

A total of 865 Israeli adults responded to the survey (sampling error +/− 3.2% at 0.95 statistical interval), and the response rate was 31%. Table 1 (Socio-demographic characteristics of the study participants) presents the raw sample data prior to the weighting, the data after standardization and the data from the Central Bureau of Statistics. In purpose to express a representative sample of the Israeli population each group as been sampled went a process of standardization to got a relative weight in the population of Israel according to data from the C.B.S. Most participants were women (51.8%), Jewish (68.6%), and married (73.0%). A quarter of the participants (162, 18.8%) were older than 65 years and 34.1% (294) were 45–64 years old. In terms of religious orientation, over half of the participants (350, 47.8%) defined themselves as traditional or religious. Most participants were first-generation Israelis (367, 50.3%) and over a quarter (198, 27.1%) were second-generation Israelis. Half of the participants (415, 48.0%) had a Elementary and high school education and another 52.0% (450) had post-secondary education. Most of the participants (728, 84.1%) reported having good to very good health; 242 (27.9%) reported a chronic illness. Considering that the average wage in Israel at the time of the study was 9800 New Israeli Shekels (NIS), about a quarter of the participants (194, 22.5%) had an income below NIS 8600, and 24.2% (244) had an income between NIS 8600 and 16,600. As with the distribution of the population, about half of the sample reported that they were registered at Clalit Health Services, about 23.7% at Maccabi and the small number of participants in the Meuhedet / Leumit health fund. The overwhelming majority of the sample (83.5%) reported that they had supplementary health insurance (SHS) through the HMO, and about half of the sample (48.3%) reported that they had private / commercial health insurance.

**Table 1:** Socio-demographic characteristics of the study participants^1^

Variables	Study population ***N*** = 865 n (%)	Distribution prior to the weighting ***N*** = 856 n (%)	Statistical data of The Central Bureau of Statistics (CBS) n (%)
**Gender**			
Male	417 (48.2%)	333 (38.5%)	48.4%
Female	448 (51.8%)	532 (61.5%)	51.6%
**Sector**			
Jewish sector (general)	593 (68.6%)	618 (71.4%)	62.9%
Jewish (Immigrants from the former Soviet Union)	138 (16.0%)	129 (14.9%)	16.0%
Arabic (Non Jewish)	134 (15.5%)	118 (13.6%)	21.1%
**Age, years**			
25–34	211 (24.4%)	68 (7.9%)	24.1%
35–44	197 (22.8%)	182 (21.0%)	22.4%
45–54	155 (18.0%)	158 (18.3%)	17.9%
55–64	139 (16.1%)	214 24.7%)	15.4%
> 65	162 (18.8%)	243 (28.1%)	20.2%
**Religious orientation** (Jewish)			
Secular	382 (52.2%)	402 (53.8%)	44.2%^2^
Traditional	209 (28.6%)	143 (19.1%)	35.1%
Religious and ultra-orthodox	141 (19.2%)	202 (27.1%)	20.2%
**Parents’ Country of Origin**			
Israel (Second generation that born in Israel)	198 (27.1%)	155 (20.7%)	54.5%^3^
Eastern Origin (countries of the basin and the eastern Mediterranean)	204 (28.0%)	213 (28.5%)	26.6%
Western Origin)Western and Eastern European countries)	163 (22.3%)	234 (48.9%)	18.8%
Did not answer	15 (1.7%)	14 (1.9%)	–
**Marital Status**			
Single	126 (14.6%)	53 (6.1%)	14.2%
Married	632 (73%)	685 (79.2%)	70.9%
Divorced/separated	38 (4.4%)	41 (4.7%)	9.2%
Widowed	41 (4.7%)	63 (7.3%)	5.7%
**Education**			
Elementary & High school	415 (48.0%)	461 (53.3%)	48.2%
Higher education	450 (52.0%)	404 (46.7%)	51.8%
**Monthly income (New Israeli Shekels)**			
> 4100 < 4000	75 (8.7%)	85 (9.8%)	7.5%
4100–6200 4–6.5	53 (6.2%)	58 (6.7%)	10.7%
6201–8600 6.5–8	66 (7.6%)	63 (7.3%)	7.1%
8601–10,900 8–10	90 (10.4%)	91 (10.5%)	10.3%
10,901–13,500 10–13	67 (7.8%)	84 (9.7%)	14.6%
13,501–16,600 13–17	87 (10.1%)	84 (9.7%)	15.3%
16,601–20,400 17–24	70 (8.1%)	70 (8.1%)	12.8%
> 20,400 24+	68 (7.8%)	73 (8.4%)	9.8%
> 45,000	12 (1.3%)	12 (1.4%)	
Did not answer	277 (32.0%)	245 (28.3%)	11.8%
**Self-rated health**			
Very good	392 (45.3%)	349 (40.3%)	51.6%
Good	336 (38.8%)	369 (42.7%)	30.7%
Not so good	95 (11.0%)	103 (11.9%)	12.8%
Not good	13 (1.5%)	18 (2.1%)	12.8%
Bad	6 (0.7%)	6 (0.7%)	4.7%
**Chronic disease** (A chronic disease is one lasting 6 months or more)	42 (27.9%)	277 (32.0%)	34.1%
**HMO membership**			
Clalit Health Services	468 (54.1%)	438 (50.6%)	52.1%
Maccabi Health Services	205 (23.7%)	201 (23.2%)	25.7%
Meuhedet	105 (12.2%)	125 (14.5%)	13.9%
Leumit	58 (6.7%)	77 (8.9%)	8.3%
Did not answer	28 (3.3%)	24 (2.8%)	–

^1^Table 1 presents the raw sample data and the data after standardization. In this study each group as been sampled and got a relative weight in the population of Israel (according to data from the Central Bureau of Statistics).

^2^CBS segmentation method: secular, religious, traditional, non-religious, religious, ultra-Orthodox.

^3^CBS Social Survey)Tested by Father’s country of origin).

### Correlation between trust in the health system, the importance of selecting a surgeon and perceived equitability in the public health system

The extent of participant agreement with each of the statements on trust, surgeon selection and perceived equitability in the public health system are summarized in Table 2. Evaluation of the percentage of respondents that expressed great to very great agreement with each statement showed that less than half of the respondents (41.0%) agreed that the public healthcare system is equitable. Over half of the respondents agreed that one has to have connections and money to receive good and beneficial medical treatment (54.4 and 57.3%, respectively). Over half of the respondents (56.2%) agreed that people who have private medical insurance or complementary health insurance receive better healthcare within the public health system. Most respondents (84.8%) agreed that the queues in the public health system are always longer than those for private healthcare, 29.6% agreed that the public health system should allow shortening queues by private pay.

**Table 2:** Participants’ perceptions regarding choice, equatability and trust in the public health system

	Study population ***N*** = 865				
**Statement**	**Agree to a very great extent**	**Agree to a great extent**	**Somewhat agree**	**Disagree**	**Do not know**
To receive good and beneficial medical treatment, one has to have connections.	n (28.9%)	n (25.5%)	n (20.7%)	n (22.8%)	n (2.1%)
To receive good and beneficial medical treatment, one has to have money**.**	n (26%)	n (31.3%)	n (18.9%)	n (23.4%)	n (0.5%)
Those who have private medical insurance or complementary health insurance receive better health care within the public health system.	n (21.3%)	n 34.9%)	n (19.9%)	n (19.3%)	n (4.6%)
In the public health system, the queues for surgery are determined according to medical justification or medical need.	n (14.3%)	n (38.8%)	n (24.5%)	n (13.6%)	n (8.7%)
The queues in the public health system are always longer than those for private healthcare**.**	n (51.5%)	n (33.3%)	n (6.6%)	n (4.0%)	n (4.6%)
I believe and trust my HMO physician.	n (27.9%)	n (44.6%)	n (18.6%)	n (8.0%)	n (0.9%)
I believe and trust the physician who attended to me in the hospital.	n (17.3%)	n (52.2%)	n (22.3%)	n (5.6%)	n (2.7%)
The public health system is equitable.	n (11.1%)	n (29.9%)	n (29.0%)	n (27.1%)	n (2.9%)
The public health system should allow shortening queues by private pay.	n (12.3%)	n (17.3%)	n (18.0%)	n (49.7%)	n (2.6%)
Everyone should be allowed to choose a surgeon, free of charge, in public hospitals.	n (48.1%)	n (33.9%)	n (10.0%)	n (6.7%)	n (1.2%)
**Statement**	**Very important**	**Important**	**Not so important**	**Not important at all**	**Do not know**
Should you require surgery, how important would it be for you to choose the hospital where the surgery will be performed?	n (64.8%)	n (27.6%)	n (4.6%)	n (2.8%)	n (0.2%)
Should you require surgery, how important would it be for you to choose the surgeon?	n (63.2%)	n (26.9%)	n (6.3%)	n (3.0%)	n (0.4%)

Over half of the respondents (53.1%) agreed that in the public health system the queues for surgery are determined according to medical justification or medical need. Most participants trust their HMO physician and hospital physician (72.5 and 69.5%, respectively), and most (82.0%) agreed that everyone should be allowed to choose a surgeon, free of charge, in public hospitals. The majority of respondents considered selecting the surgeon and the hospital for surgery as important to very important (90.1 and 92.4%. respectively).

### Association between perceptions of trust, the importance of selecting a surgeon and equitability of the public health system

Table 3 shows the results of Pearson’s correlation between statements on “perception of the equitability of the public health system” “trust in the health system” and “the importance of selecting a surgeon”. Significant positive correlations were found between all the statements that comprise the variable “perception of the health system as inequitable” and the statements that comprise the variable “importance of selecting a surgeon.” Thus, a higher rate of support for statements that express the perception of the system as inequitable, correspond with a higher rate of support for the need to select a surgeon. In addition, significant correlations were found between most of the statements that comprise the variable “perception of the health system as inequitable” and between “trust in the health system”.

**Table 3:** Pearson correlation between trust, physician selection, and equality in the public health system

	Study population ***N*** = 865	
Statements on the perception of the system as inequitable	Trust r (***p*** value)	Choosing a surgeon r (***p*** value)
To receive good and beneficial medical treatment, one has to have connections.	−0.24 (< 0.01)	0.21 (< 0.01)
To receive good and beneficial medical treatment, one has to have money**.**	−0.19 (< 0.01)	0.21 (< 0.01)
The public health system is equitable.	0.61 (< 0.01)	- 0.11 (< 0.01)
Those who have private medical insurance or complementary health insurance receive better health care within the public health system.	0.02(NS)	0.20 (< 0.01)
The queues in the public health system are always longer than those for private healthcare**.**	−0.04 (NS)	0.13 (< 0.01)
The public health system should allow shortening queues by private pay.	0.05(NS)	0.08 (< 0.05)

Grouping of these statements showed that statements relating to “trust in the public health system” were negatively correlated with statements relating to the “importance of selecting a surgeon” (*r* = − 0.22, *p* < 0.01) and with statements relating to “the perception that the health system is inequitable” (*r* = 0.61, *p* < 0.01). In other words, the.

higher the level of trust in the public health system, the less it is perceived as less important to select a surgeon and vice versa. In addition, the higher the level of trust in the public health system, the higher the system is perceived as higher equability and vice versa.

The “perception that the health system is inequitable” had a significant positive correlation with the “importance of selecting a surgeon” (*r* = − 0.11, *p* < 0.01). Hence, the more the public health system is perceived as inequitable, it is perceived as higher important to select a surgeon.

### Variables predicting “trust in the public health system”

Multivariate linear regression analysis was used to determine which socio-demographic background variables contributed to predicting “trust in the public health system”. As shown in Table 4, the final model included nine independent predictor variables that significantly explained 13% of the observed variance of trust in the public health system (F (10, 831) = 12.12, *p* < 0.01). Notably, “membership in Leumit Health Care Services”, “membership in Kupat Holim Meuhedet”, and “health status” were introduced into the initial model but their contribution was not statistically significant and therefore they were not included in the final model. The regression coefficients indicate that “education”, belonging to the “Jewish Sector”, belonging to the “Jewish immigrants from the former Soviet Union” sector, and “membership in Clalit Health Services” significantly contributed to the model. Hence, higher education and a membership in Clalit Health Services were associated with a higher level of trust, while belonging to the Jewish Sector and being a Jewish immigrant from the former Soviet Union were associated with a lower level of trust (compared to the Arab sector).

**Table 4:** Regression coefficients for predicting trust in the public health system

Predictors		Coefficients			
	***β***	***SE***	***B***	t	*R*^*2*^
Gender (male)	0.01	0.04	0.01	0.34	
Age	0.03	0.01	0.08	2.10*	0.13
Marital status (married)	0.06	0.04	0.05	1.39	
Education	0.03	0.01	0.09	2.80**	
Income	−0.01	0.01	− 0.04	−1.12	
Jewish sector (general)	−0.50	0.06	−0.41	−8.88**	
Jewish sector (immigrants from the former Soviet Union)	−0.34	0.07	−0.21	−4.65**	
HMO (Clalit Health Services)	0.13	0.05	0.12	2.72**	
HMO (Maccabi Healthcare Services)	0.17	0.06	0.12	2.97**	

*HMO* Health maintenance organization

*p < 0.05; **p < 0.01

### Variables predicting the perception of the public health system as inequitable

To determine which variables contribute to the “perception of the public health system as inequitable”, hierarchical linear regression was performed. Socio-demographic variables were introduced in the first step, and “trust in the public health system” was introduced in the second step. As shown in Table 5, the final model included 7 independent predictor variables that significantly explained 6% of the observed variance in “perception of the health system as inequitable” (F (7, 834) = 8.12, *p* < 0.01). Notably, “income”, “education”, “age”, and “membership in Maccabi Healthcare Services”, “membership in Leumit Health Care Services” or “membership in Kupat Holim Meuhedet”, were introduced into the initial model but their contribution was not statistically significant and therefore they were not included in the final model.

**Table 5:** Regression coefficients for predicting the perception of the health system as inequitable

Predictors		Coefficients			
	***β***	***SE***	***B***	t	*R*^*2*^
**First step**					0.06
Gender (male)	− 0.01	0.04	− 0.01	−0.13	
Marital status (married)	0.09	0.05	0.06	1.78	
Health status	0.05	0.03	0.06	1.61	
Jewish sector (general)	0.37	0.06	0.26	6.01**	
Jewish sector (immigrants from the former Soviet Union)	0.13	0.08	0.07	1.58	
HMO (Clalit Health Services)	− 0.03	0.05	− 0.02	− 0.68	
**Second step**					
Trust	−0.10	0.04	−0.09	−2.45**	0.06

*HMO* Health maintenance organization

*p < 0.05; **p < 0.01

The regression coefficients indicated that “trust in the public health system” and “belonging to the Jewish sector” significantly contributed to the model. In other words, belonging to the Jewish sector and less trust in the public health system were significantly associated with the perception that the health system is more inequitable.

### Variables predicting the importance of selecting a surgeon

Hierarchical linear regression was performed to predict “the importance of selecting a surgeon”. Socio-demographic variables were introduced in the first step. “Trust in the public health system” and “the perception of the health system as inequitable” were introduced in the second step. As shown in Table 6, the final model included 12 independent predictor variables that significantly explained 13% of the observed variance in predicting the importance of surgeon selection (F (12, 829) = 10.61, *p* < 0.01). Notably, “membership in Leumit Health Care Services” or “membership in Kupat Holim Meuhedet”, were introduced into the initial model but their contribution was not statistically significant and therefore they were not included in the final model.

**Table 6:** Regression coefficients for predicting the importance of surgeon selection

Predictors		Coefficients			
	***β***	***SE***	***B***	t	*R*^*2*^
**First step**					0.06
Gender (male)	− 0.01	0.04	− 0.01	− 0.77	
Age	−0.03	0.02	−0.01	−1.89	
Marital status (married)	0.07	0.05	0.05	1.45	
Education	−0.01	0.01	−0.03	− 0.75	
Income	0.01	0.01	0.04	0.94	
Health status	−0.01	0.03	−0.01	− 0.30	
Jewish sector (general)	0.32	0.06	0.26	5.43**	
Jewish sector (immigrants from the former Soviet Union)	0.15	0.08	0.09	1.92	
HMO (Clalit Health Services)	−0.12	0.05	−0.10	−2.42*	
HMO (Maccabi Healthcare Services)	−0.12	0.06	−0.09	−2.08*	
**Second step**					
Trust	−0.14	0.04	− 0.14	−3.87**	0.13
Inequability	0.21	0.03	0.23	6.91**	

*HMO* Health maintenance organization

*p < 0.05; **p < 0.01

The regression coefficients indicated that “income”, “belonging to the Jewish sector”, “membership in Clalit Health Services”, “the perception of the health system as inequitable”, and “trust in the public health system” made a statistically significant contribution to the model. In other words, belonging to the Jewish sector, having a higher income and perceiving the health system as more inequitable were associated with attributing greater importance to selecting a surgeon, while membership in Clalit Health Care Services and a higher level of trust in the public health system were related to attributing less importance to selecting a surgeon.

### Predicting the importance of selecting a surgeon based on trust in the public health system and perceiving it as inequitable

To examine the possibility that the “perception of the public health system as inequitable” mediate the association between “trust in the public system” and the “importance of selecting a surgeon”, a hierarchical regression analysis was performed. As shown in Table 7, in the first step, “trust in the public health system” was introduced into the model as the independent variable. In the second step, the “perception of the health system as inequitable” was also introduced into the model. The model was statistically significant (F (2, 862) = 56.11, *p* < 0.01). The analysis showed that “trust in the public health system” has a unique and statistically negative contribution, accounting for 5% of the observed variance to the “importance of selecting a surgeon”. In the second step, the influence of “trust in the public health system” becomes weaker, while the “perception of the public health system as inequitable” predicts a further 7% to explaining the difference of the “importance of selecting a surgeon”. This analysis indicates that the “perception of the health system as inequitable” mediates the relationship between “trust in the public health system” and the “importance of selecting a surgeon”.

**Table 7:** Regression coefficients for predicting the importance of surgeon selection according to trust and perception of equability

Predictors		Coefficients			
	***β***	***SE***	***B***	t	*R*^*2*^
**First step**					
Trust	−0.23	0.03	−0.22	−6.69**	0.05
**Second step**					
Trust	−0.18	0.03	−0.18	−5.60**	0.12
Equability	0.23	0.03	0.26	8.00**	

**p < 0.01

## Discussion

The importance of the National Health Insurance Law lies in its defining principles: justice, equability and mutual assistance. These three principles determine the ideals and the service benchmarks set by the public health system. The survey results emphasize that there is a possible association between three meaningful factors: the trust in the public health system, the perception of the system’s equitability and the public’s perception of the importance of selecting a surgeon. This study did not examine the causal relationship between the various factors, but the study data suggests a possible connection between lower trust in the system and lower perception of its equitability, and a subsequent associated increase in the public’s desire to select a surgeon and thus to have an influence on their choice of service provider. The public’s confidence in the health system has been identified and marked as a significant and critical factor by the public whose views are reflected in the research data (Note: reference to the issue of agreements at the limitations section) .

It seems that after three decades during which senior policymakers have attempted to warn the government and health investors that the public health system is “dying” and/or “collapsing,” this message perhaps has permeated the public, and influenced its perceptions and contributed to its growing distrust of the public health system’s capabilities. In other words, it seems that this perception came from “above” to the Israeli public, rather than from the people to the policymakers. As such distrust has a systemic impact on the health system, it should be defined as the most significant and critical problem of the public health system.

Support for the issue of public trust in the health system and its various implications can be found in several surveys conducted over the last few years. For example, in a survey conducted by the Brookdale Institute, 84% of the respondents claimed that it was necessary to use personal and/or professional connections in order to receive good treatments quickly during a serious illness [13]. These reports are similar to the findings of a corresponding study in which 54% of the respondents reported that they are “not sure” or “not so sure” that in case of a serious illness they would receive the best and most effective treatment in the Public health system [13]. In another survey that examined the public’s attitudes towards health services in Israel, only 39.5% of the respondents expressed their trust in the health system, reflecting “a doubt that exists among the public regarding the ability of the public health system to provide an appropriate response.” Similar results have been shown in other studies that each group of non-Jews (Muslims, Christians, Druze) believes that the healthcare system has a great deal of equality (38%+), compared to only 28% of Jews [11, 14]. Similar results were also found in this survey (group of “Jewish” being significantly correlated with a view of system as being inequitable). The survey also found significant differences between the public’s image of the family physician (community medicine) and the image of hospital staff. The respondents expressed greater trust in their HMOs than in the hospitals [6]. similar results have been shown in other studies [3, 11]. As stated, these data correspond to data that underlie this work, and they can reinforce the findings and conclusions.

Another manifestation of the crisis in the public health system is reflected in the percentage of individuals who do not believe that they would be able to rely on the public health system in times of need, and therefore purchase complementary or commercial health insurance (75 and 40% respectively) [7]. These data are in accordance with a survey conducted in 2017, in which 84% of respondents reported ownership of any complementary health insurance, and 57% reported ownership of any commercial health insurance policy. The authors of the report also noted that 52% of the respondents had both supplemental and commercial health insurance, while only 11% had no additional health insurance to the basic health basket [3]. Similarly, the findings of this study showed that a high rate of respondents had supplementary insurance (82.5%) and commercial insurance (49.8%). While some see the accelerated rate of private insurance holders as an expression of free will, rational preference of risk and taking personal responsibility for health, others see it as an erosion of the public health basket and the continuing damage to its availability and quality, which has led to lower trust in the public health system and to a search for an alternative private insurance coverage [15]. This is evidence for the effects of reduced ‘public trust’ on the health system’s effectiveness, its functioning, and the realization of its goals.

In light of the public health system’s limited resources and the fear of violating the principle of equality that stands at the heart of the National Insurance Law, it seems that the key to maintaining the public health system is to enhance the public’s trust in the system as a whole, and particularly in its efficiency and various mechanisms.

The distrust in the public health system is associated in the public’s perception with the importance of selecting a physician, and particularly with selecting a surgeon in public hospitals. The ability to select a surgeon in a public hospital is the means by which the public tries to minimize the level of uncertainty, and to take more control over decisions regarding its own health and life. Here is the place to note the trade-off that can exist between surgeon choice and waiting time. For example, allowing surgeon choice can cause a long wait time for that particular surgeon; so the patient has to choose between waiting time / choosing a surgeon, in correlation to his own preferences and situation.

The findings of the study suggest that it could be useful to consider expanding the range of choices available to the public within the health system in a proportionate, responsible and informed manner while ensuring that the principle of equality is implemented in the allocation of resources in the health system.

This study is a sample of 865 participants representing all sectors of Israeli society and thus providing an accurate picture of the current perceptions about the public health system.

### The limitations of the study include

1. The research findings reflect attitudes, perceptions and opinions that are correct for the day of the study (Note: The common phrase ‘Agreed’ expresses the degree of consent with the a certain assumption/ statement, but it does not indicate that the assumption/statement is a fact. Also, “agreed” can be used by some respondents as a default response).
2. The findings of the quantitative research express public attitudes and perceptions as reported in a telephone survey. The rate of response to the telephone survey is low (31%), which may affect the degree of representation of the sample. The low response rate is explained in the scientific literature as a result of a variety of factors and processes that have occurred over the years. (Eg, between women and men), based on social perception (decreased willingness to respond to surveys) and technological progress (identifying and ignoring unidentified telephone numbers). In order to deal with the low response rate, and to purpose to express a representative sample of the Israeli population each group as been sampled went a process of standardization to got a relative weight in the population of Israel according to data from the C.B.S.
3. Quantitative research findings represent the observed variables, but due to limitations of the study design (a cross-sectional analysis), the statistical correlations found do not necessarily reflect causal relationships.
  In order to strengthen the internal validity and the external validity of the research, an integrated research methodology was used, which included constructing a questionnaire based on qualitative research findings, piloting of the interviews, statistical analysis in a number of methods (univariate, multivariate, Indices, etc.).

## Conclusions

Israeli policymakers believe that public trust in the public health system is a fundamental condition for maintaining an efficient and equitable health system in Israel [12]. The survey of the general public suggests that uncertainty regarding the identity of the surgeon who will perform a procedure in a public hospital may be linked to a sense of insecurity and distrust of the public in the public health system. The survey also suggests that this may, in turn, encourage the public to turn to the private health system in which the individual has more control over choosing the treating surgeon.

Therefore, it may be that promoting public trust in the public health system could be a mechanism for reducing the usage of the private health system, decreasing private health expenditure in the long run. One of the ways to strengthen the public’s confidence in the public health system could be to provide the patient with reliable information regarding parameters such as the identity of the senior surgeon in the operating room or the surgeon’s suitability for the patient’s medical condition. The possibility should be accessible to everyone, procedurally and substantially - which means providing reliable information and tools for informed choice by hospital staff, free of charge from the patient or anyone acting on his behalf, etc. These possibilities should be further explored in future studies, that would be designed to more fully assess causal connections (including what the effect of media contribution to lowering trust in the public health system).

The findings of this survey can help policymakers recognized that a key element in achieving balance and regulation of fulfillment in the public health system lies in a significant and decisive component, which is, public trust in the public health system, its efficiency and its various mechanisms. Public trust through which it is possible to reduce the potential risk of inefficient or improper use of public resources in the health system.

## Data Availability

The authors are not able to share the data in order to preserve the privacy of the respondents.
